# *trans*-Dichloridotetra­pyrazine­ruthenium(II) dichloro­methane disolvate

**DOI:** 10.1107/S1600536812035817

**Published:** 2012-08-23

**Authors:** Vladimir N. Nesterov, Wajiha Khan, Alexandra E. Rangel, Bradley W. Smucker

**Affiliations:** a Department of Chemistry, University of North Texas, 1155 Union Circle, #305070, Denton, TX 76203-5070, USA; b Department of Chemistry, Austin College, 900 North Grand, Sherman, TX 75090-4400, USA

## Abstract

In the title compound, [RuCl_2_(C_4_H_4_N_2_)_4_]·2CH_2_Cl_2_, the Ru^II^ atom occupies a position of 222 symmetry and the C atom of the solvent mol­ecule occupies a site with twofold symmetry. The Ru^II^ atom has a slightly distorted octa­hedral geometry. The pyrazine rings are propeller-like and rotated 45.1 (1)° from the RuN_4_ plane. In the crystal, the complex and solvent mol­ecules are bridged by weak C—H⋯N hydrogen bonds along the *c* axis. Weak inter­molecular C—H⋯Cl contacts link the complexes in the *ab* plane, forming a network.

## Related literature

The synthesis of the title complex and its use as a building block in coordination networks are described by Carlucci *et al.* (2002 ▶) and Coe (2004 ▶). For related structures using pyridine and varying *trans* ligands, see: Coe *et al.* (1995 ▶); Desjardins *et al.* (1999 ▶).
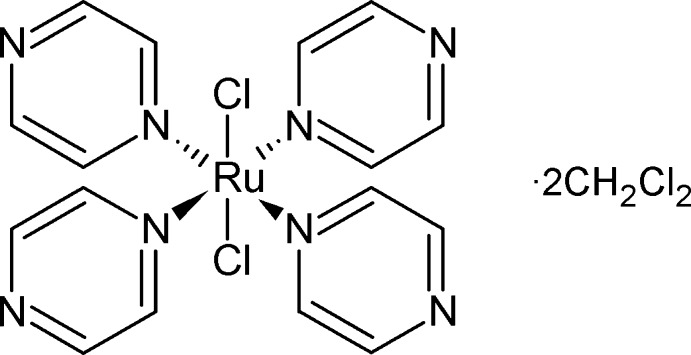


## Experimental

### 

#### Crystal data

[RuCl_2_(C_4_H_4_N_2_)_4_]·2CH_2_Cl_2_
*M* *_r_* = 662.19
Tetragonal, 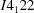

*a* = 7.3059 (2) Å
*c* = 47.3659 (16) Å
*V* = 2528.21 (14) Å^3^
*Z* = 4
Mo *K*α radiation
μ = 1.28 mm^−1^
*T* = 100 K
0.10 × 0.10 × 0.08 mm

#### Data collection

Bruker APEXII CCD diffractometer
Absorption correction: multi-scan (*SADABS*; Bruker, 1996 ▶) *T*_min_ = 0.882, *T*_max_ = 0.908
15409 measured reflections
1399 independent reflections
1363 reflections with *I* > 2σ(*I*)
*R*_int_ = 0.030

#### Refinement

*R*[*F* ^2^ > 2σ(*F* ^2^)] = 0.016
*wR*(*F* ^2^) = 0.040
*S* = 1.03
1399 reflections
81 parameters
H atoms treated by a mixture of independent and constrained refinement
Δρ_max_ = 1.01 e Å^−3^
Δρ_min_ = −0.38 e Å^−3^
Absolute structure: Flack (1983 ▶), 508 Friedel pairs
Flack parameter: 0.26 (4)

### 

Data collection: *APEX2* (Bruker, 2007 ▶); cell refinement: *SAINT* (Bruker, 2007 ▶); data reduction: *SAINT*; program(s) used to solve structure: *SHELXS97* (Sheldrick, 2008 ▶); program(s) used to refine structure: *SHELXL97* (Sheldrick, 2008 ▶); molecular graphics: *SHELXTL* (Sheldrick, 2008 ▶); software used to prepare material for publication: *SHELXTL*.

## Supplementary Material

Crystal structure: contains datablock(s) I, global. DOI: 10.1107/S1600536812035817/tk5139sup1.cif

Structure factors: contains datablock(s) I. DOI: 10.1107/S1600536812035817/tk5139Isup2.hkl

Supplementary material file. DOI: 10.1107/S1600536812035817/tk5139Isup3.mol

Additional supplementary materials:  crystallographic information; 3D view; checkCIF report

## Figures and Tables

**Table 1:** Hydrogen-bond geometry (Å, °)

*D*—H⋯*A*	*D*—H	H⋯*A*	*D*⋯*A*	*D*—H⋯*A*
C3—H3*A*⋯Cl1^i^	0.95	2.88	3.555 (2)	129
C5—H5*A*⋯N2^ii^	0.92 (2)	2.46 (2)	3.338 (2)	158 (2)

Symmetry codes: (i) 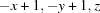; (ii) 
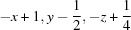
.

**Fig. 1.**
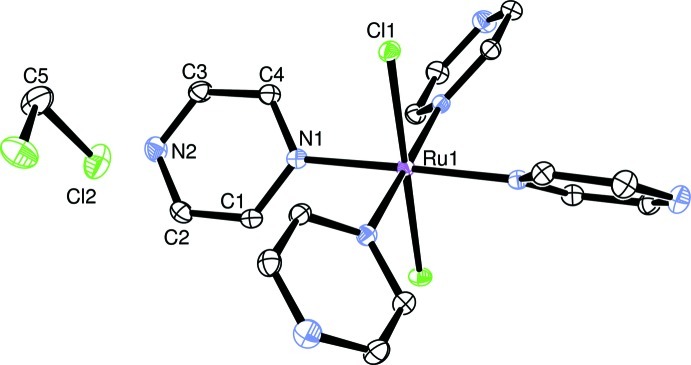
View of the title compound with 50% probability displacement ellipsoids.

**Fig. 2.**
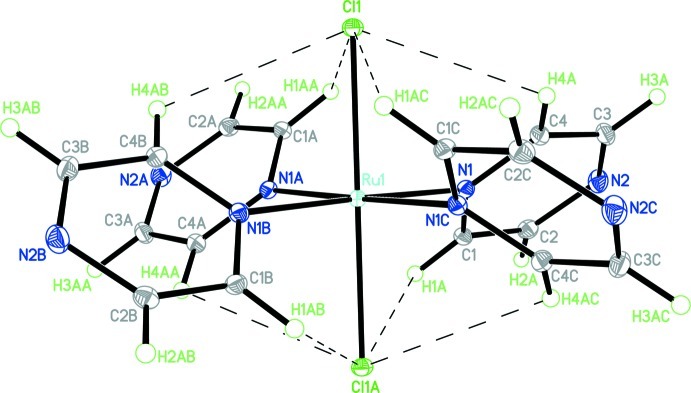
View of the title compound showing intramolecular Cl···H contacts.

**Fig. 3.**
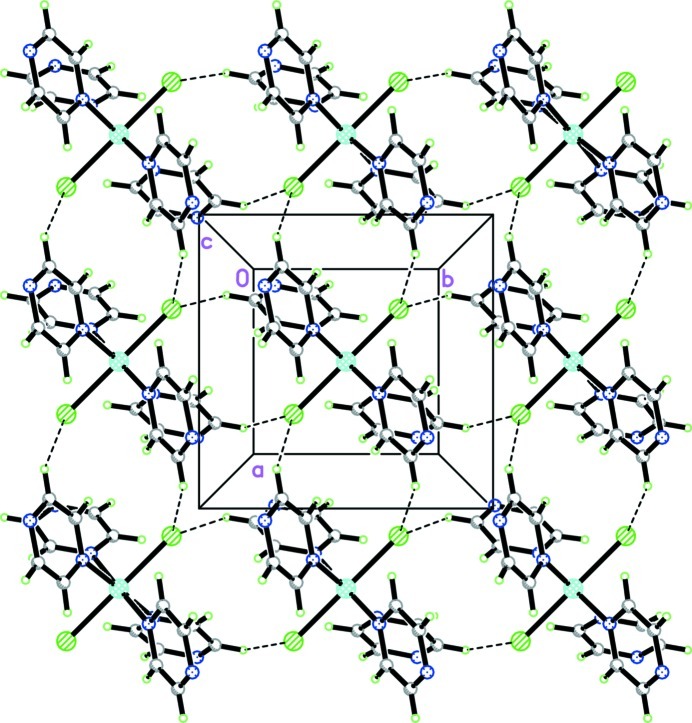
Fragment of the crystal packing of the compound along a c axis (dashed lines are Cl···H contacts).

**Fig. 4.**
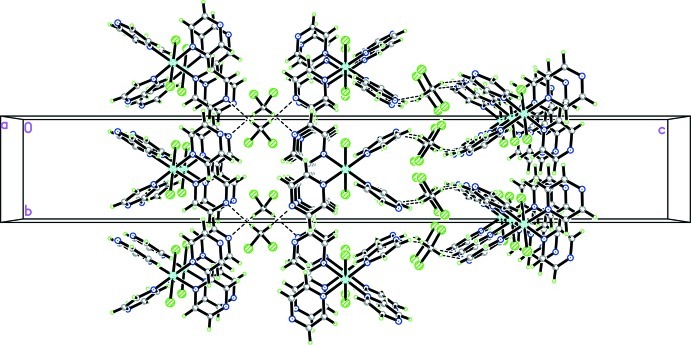
Fragment of the crystal packing of the compound along an a axis (dashed lines are C—H···N H-bonds).

## supplementary crystallographic information

### Comment

The pyrazine ligands are rotated 45.1 (1)° from the N—Ru—N plane (Fig. 1) consistent with other propeller-like structures (Coe *et al.*, 1995 and references therein). The terminal chloride atoms on the Ru(pz)_4_Cl_2_ complexes are 2.86 - 2.94 Å from the four hydrogen atoms belonging to neighboring pyrazine groups (Fig. 2). This additional interaction enhances the stability of the propeller-like structure.

The Ru—Cl bond length is 2.3920 (5) Å and the Ru—N bond length is 2.0620 (14) Å. These distances are on the low side of the narrow range of bond lengths when this complex is used in supramolecular assemblies (Carlucci *et al.*, 2002), indicating very little influence on bond distance upon further coordination of this metal-based building block. Ru—N distances in tetrakis(pyridine)Ru*L*_2_, are 2.09 Å (*L* = 2-chlorophenylcyanamide) (Desjardins, *et al.*, 1999), or 2.08 Å (*L* = one chloride and one benzonitrile) (Coe *et al.*, 1995).

H-bonds and intermolecular contacts form a network in the crystal. Atom Cl1 has an intermolecular contact with a hydrogen atom on two pyrazine ligands on a neighboring complex (Fig. 3 and Table 1). At the same time, the hydrogen atoms of the dichloromethane solvate have weak hydrogen bonds between two terminal N-atoms on the pyrazine ligands of two separate Ru(pz)_4_Cl_2_ complexes (Fig. 4 and Table 1). Additionally, the solvent chloride atom is 3.383 (3) Å from the C2 atom near the uncoordinated nitrogen on the pyrazine ligand.

### Experimental

The ruthenium complex was synthesized by the student co-authors in the laboratory component of Austin College's advanced inorganic course according to procedures by Carlucci *et al.* (2002) and Coe (2004). Crystals of the title compound were grown from a slow diffusion of hexanes into a solution of the ruthenium complex dissolved in dichloromethane.

### Refinement

The H atoms attached to C atoms of the pyrazine molecules were placed in idealized positions (C—H = 0.95 Å) and allowed to ride on their parent atoms. Their positions were constrained so that the *U*_iso_(H) was equal to 1.2*U*_eq_ of their respective parent atoms. The solvent molecule, CH_2_Cl_2_, occupies a special position in the unit cell so the H atom was located using a difference map and was refined with a constrained *U*_iso_(H) equal to 1.2*U*_eq_ of its parent atom.

The maximum and minimum residual electron density peaks of 1.01 and 0.37 eÅ^-3^, respectively, were located 1.44 Å and 0.76 Å from the H4A and Ru1 atoms, respectively, with the large residue most likely due to imperfect absorption corrections frequently encountered in heavy-metal atom structures.

### Crystal data

**Table:**

[RuCl_2_(C_4_H_4_N_2_)_4_]·2CH_2_Cl_2_	*D*_x_ = 1.740 Mg m^−^^3^
*M**_r_* = 662.19	Mo *K*α radiation, λ = 0.71073 Å
Tetragonal, *I*4_1_22	Cell parameters from 8671 reflections
Hall symbol: I 4bw 2bw	θ = 2.8–27.0°
*a* = 7.3059 (2) Å	µ = 1.28 mm^−^^1^
*c* = 47.3659 (16) Å	*T* = 100 K
*V* = 2528.21 (14) Å^3^	Plate, black
*Z* = 4	0.10 × 0.10 × 0.08 mm
*F*(000) = 1320	

### Data collection

**Table:**

Bruker APEXII CCD diffractometer	1399 independent reflections
Radiation source: fine-focus sealed tube	1363 reflections with *I* > 2σ(*I*)
Graphite monochromator	*R*_int_ = 0.030
ω scans	θ_max_ = 27.0°, θ_min_ = 1.7°
Absorption correction: multi-scan (*SADABS*; Bruker, 1996)	*h* = −9→9
*T*_min_ = 0.882, *T*_max_ = 0.908	*k* = −9→9
15409 measured reflections	*l* = −60→60

### Refinement

**Table:**

Refinement on *F*^2^	Secondary atom site location: difference Fourier map
Least-squares matrix: full	Hydrogen site location: inferred from neighbouring sites
*R*[*F*^2^ > 2σ(*F*^2^)] = 0.016	H atoms treated by a mixture of independent and constrained refinement
*wR*(*F*^2^) = 0.040	*w* = 1/[σ^2^(*F*_o_^2^) + (0.022*P*)^2^ + 2.540*P*] where *P* = (*F*_o_^2^ + 2*F*_c_^2^)/3
*S* = 1.03	(Δ/σ)_max_ = 0.001
1399 reflections	Δρ_max_ = 1.01 e Å^−^^3^
81 parameters	Δρ_min_ = −0.38 e Å^−^^3^
0 restraints	Absolute structure: Flack (1983), 508 Friedel pairs
Primary atom site location: structure-invariant direct methods	Flack parameter: 0.26 (4)

### Special details

**Table:**

Geometry. All e.s.d.'s (except the e.s.d. in the dihedral angle between two l.s. planes) are estimated using the full covariance matrix. The cell e.s.d.'s are taken into account individually in the estimation of e.s.d.'s in distances, angles and torsion angles; correlations between e.s.d.'s in cell parameters are only used when they are defined by crystal symmetry. An approximate (isotropic) treatment of cell e.s.d.'s is used for estimating e.s.d.'s involving l.s. planes.
Refinement. Refinement of *F*^2^ against ALL reflections. The weighted *R*-factor *wR* and goodness of fit *S* are based on *F*^2^, conventional *R*-factors *R* are based on *F*, with *F* set to zero for negative *F*^2^. The threshold expression of *F*^2^ > σ(*F*^2^) is used only for calculating *R*-factors(gt) *etc*. and is not relevant to the choice of reflections for refinement. *R*-factors based on *F*^2^ are statistically about twice as large as those based on *F*, and *R*- factors based on ALL data will be even larger.

### Fractional atomic coordinates and isotropic or equivalent isotropic displacement parameters (Å^2^)

**Table:**

	*x*	*y*	*z*	*U*_iso_*/*U*_eq_	
Ru1	1.0000	0.5000	0.2500	0.01016 (7)	
Cl1	0.76849 (5)	0.26849 (5)	0.2500	0.01419 (11)	
N1	0.8611 (2)	0.6437 (2)	0.21927 (3)	0.0125 (3)	
C1	0.9492 (2)	0.7136 (2)	0.19664 (3)	0.0140 (3)	
H1A	1.0775	0.6961	0.1949	0.017*	
Cl2	0.74959 (8)	0.41921 (8)	0.141441 (10)	0.02798 (12)	
N2	0.6770 (2)	0.8453 (2)	0.17735 (3)	0.0194 (3)	
C2	0.8562 (2)	0.8101 (2)	0.17601 (4)	0.0163 (4)	
H2A	0.9230	0.8534	0.1601	0.020*	
C3	0.5910 (3)	0.7769 (3)	0.19991 (4)	0.0178 (4)	
H3A	0.4634	0.7987	0.2019	0.021*	
C4	0.6795 (3)	0.6757 (2)	0.22056 (3)	0.0146 (3)	
H4A	0.6108	0.6279	0.2359	0.018*	
C5	0.6136 (4)	0.2500	0.1250	0.0271 (6)	
H5A	0.546 (3)	0.309 (3)	0.1112 (4)	0.033*	

### Atomic displacement parameters (Å^2^)

**Table:**

	*U*^11^	*U*^22^	*U*^33^	*U*^12^	*U*^13^	*U*^23^
Ru1	0.00969 (8)	0.00969 (8)	0.01110 (12)	0.00041 (10)	0.000	0.000
Cl1	0.01315 (16)	0.01315 (16)	0.0163 (2)	−0.0024 (2)	−0.00218 (17)	0.00218 (17)
N1	0.0128 (8)	0.0111 (7)	0.0134 (6)	0.0000 (5)	0.0006 (6)	−0.0012 (6)
C1	0.0121 (8)	0.0141 (8)	0.0159 (8)	−0.0009 (6)	0.0015 (6)	−0.0008 (7)
Cl2	0.0296 (3)	0.0277 (3)	0.0266 (3)	−0.0024 (2)	−0.0107 (2)	−0.0016 (2)
N2	0.0207 (8)	0.0184 (8)	0.0191 (7)	0.0031 (6)	−0.0011 (7)	0.0044 (6)
C2	0.0187 (9)	0.0150 (9)	0.0153 (8)	−0.0017 (7)	0.0023 (7)	0.0015 (7)
C3	0.0133 (9)	0.0194 (9)	0.0207 (9)	0.0024 (7)	0.0013 (7)	0.0011 (7)
C4	0.0135 (9)	0.0147 (9)	0.0157 (7)	0.0000 (6)	0.0011 (7)	0.0005 (7)
C5	0.0182 (14)	0.0382 (19)	0.0249 (14)	0.000	0.000	−0.0117 (14)

### Geometric parameters (Å, º)

**Table:**

Ru1—N1	2.0620 (14)	Cl2—C5	1.7668 (17)
Ru1—N1^i^	2.0620 (14)	N2—C2	1.336 (2)
Ru1—N1^ii^	2.0620 (14)	N2—C3	1.337 (2)
Ru1—N1^iii^	2.0620 (14)	C2—H2A	0.9500
Ru1—Cl1^iii^	2.3920 (5)	C3—C4	1.386 (3)
Ru1—Cl1	2.3920 (5)	C3—H3A	0.9500
N1—C4	1.349 (2)	C4—H4A	0.9500
N1—C1	1.351 (2)	C5—Cl2^iv^	1.7668 (17)
C1—C2	1.383 (2)	C5—H5A	0.92 (2)
C1—H1A	0.9500		
			
N1—Ru1—N1^i^	89.82 (8)	C1—N1—Ru1	121.20 (12)
N1—Ru1—N1^ii^	178.62 (9)	N1—C1—C2	121.29 (16)
N1^i^—Ru1—N1^ii^	90.20 (7)	N1—C1—H1A	119.4
N1—Ru1—N1^iii^	90.20 (7)	C2—C1—H1A	119.4
N1^i^—Ru1—N1^iii^	178.62 (9)	C2—N2—C3	115.27 (16)
N1^ii^—Ru1—N1^iii^	89.82 (7)	N2—C2—C1	123.07 (17)
N1—Ru1—Cl1^iii^	89.31 (5)	N2—C2—H2A	118.5
N1^i^—Ru1—Cl1^iii^	90.69 (5)	C1—C2—H2A	118.5
N1^ii^—Ru1—Cl1^iii^	89.31 (5)	N2—C3—C4	122.98 (17)
N1^iii^—Ru1—Cl1^iii^	90.69 (5)	N2—C3—H3A	118.5
N1—Ru1—Cl1	90.69 (5)	C4—C3—H3A	118.5
N1^i^—Ru1—Cl1	89.31 (5)	N1—C4—C3	121.27 (16)
N1^ii^—Ru1—Cl1	90.69 (5)	N1—C4—H4A	119.4
N1^iii^—Ru1—Cl1	89.31 (5)	C3—C4—H4A	119.4
Cl1^iii^—Ru1—Cl1	180.0	Cl2—C5—Cl2^iv^	111.58 (16)
C4—N1—C1	116.08 (15)	Cl2—C5—H5A	106.7 (15)
C4—N1—Ru1	122.71 (12)	Cl2^iv^—C5—H5A	108.1 (15)

Symmetry codes: (i) −*y*+3/2, −*x*+3/2, −*z*+1/2; (ii) *y*+1/2, *x*−1/2, −*z*+1/2; (iii) −*x*+2, −*y*+1, *z*; (iv) *x*, −*y*+1/2, −*z*+1/4.

### Hydrogen-bond geometry (Å, º)

**Table:**

*D*—H···*A*	*D*—H	H···*A*	*D*···*A*	*D*—H···*A*
C3—H3*A*···Cl1^v^	0.95	2.88	3.555 (2)	129
C5—H5*A*···N2^vi^	0.92 (2)	2.46 (2)	3.338 (2)	158 (2)

Symmetry codes: (v) −*x*+1, −*y*+1, *z*; (vi) −*x*+1, *y*−1/2, −*z*+1/4.
